# Psychophysical Investigations into the Role of Low-Threshold C Fibres in Non-Painful Affective Processing and Pain Modulation

**DOI:** 10.1371/journal.pone.0138299

**Published:** 2015-09-15

**Authors:** Sumaiya Shaikh, Saad S. Nagi, Francis McGlone, David A. Mahns

**Affiliations:** 1 School of Medicine, University of Western Sydney, Sydney, NSW, Australia; 2 School of Natural Sciences and Psychology, Liverpool John Moores University, Liverpool, United Kingdom; University of Chicago, UNITED STATES

## Abstract

We recently showed that C low-threshold mechanoreceptors (CLTMRs) contribute to *touch-evoked pain* (allodynia) during experimental muscle pain. Conversely, in absence of ongoing pain, the activation of CLTMRs has been shown to correlate with a diffuse sensation of *pleasant touch*. In this study, we evaluated (1) the primary afferent fibre types contributing to positive (pleasant) and negative (unpleasant) affective touch and (2) the effects of tactile stimuli on tonic muscle pain by varying affective attributes and frequency parameters. Psychophysical observations were made in 10 healthy participants. Two types of test stimuli were applied: stroking stimulus using velvet or sandpaper at speeds of 0.1, 1.0 and 10.0 cm/s; focal vibrotactile stimulus at low (20 Hz) or high (200 Hz) frequency. These stimuli were applied in the normal condition (*i*.*e*. no experimental pain) and following the induction of muscle pain by infusing hypertonic saline (5%) into the tibialis anterior muscle. These observations were repeated following the conduction block of myelinated fibres by compression of sciatic nerve. In absence of muscle pain, all participants reliably linked velvet-stroking to pleasantness and sandpaper-stroking to unpleasantness (no pain). Likewise, low-frequency vibration was linked to pleasantness and high-frequency vibration to unpleasantness. During muscle pain, the application of previously pleasant stimuli resulted in overall pain relief, whereas the application of previously unpleasant stimuli resulted in overall pain intensification. These effects were significant, reproducible and persisted following the blockade of myelinated fibres. Taken together, these findings suggest the role of low-threshold C fibres in affective and pain processing. Furthermore, these observations suggest that temporal coding need not be limited to discriminative aspects of tactile processing, but may contribute to affective attributes, which in turn predispose individual responses towards excitatory or inhibitory modulation of pain.

## Introduction

It is widely appreciated that large myelinated mechano-afferents subserve the sensory-discriminative facet of touch, which includes pressure, vibration/texture, stretch and movement of hair follicles. In addition to the well-studied aspects of discriminative touch, there exists a distinct and independently variable affective quality of tactile sensation that contributes to our emotional response to touch [1,2]. However, it is the more commonly recognised affective dimensions of pain that have been the main focus of research with affective touch having drawn relatively little interest over the years [3–5]. Affect, an inherently subjective process, can manifest in different ways across individuals, even amongst those with previously similar sensory experiences. The inter-individual differences could be attributed to the manner in which individuals perceive a particular sensation, and whether it enhances or diminishes the link with other cognitive (e.g. fear, tension, etc.) and associated autonomic events that could be shaped by past experiences and perceived implications of an existing event [6,7].

Microneurography studies have demonstrated a class of C low-threshold mechanoreceptors (CLTMRs) in the human skin (N.B. In this paper, the abbreviation ‘CLTMRs’ refers to the C low-threshold mechanoreceptors found in a myriad of species, including *C-tactile fibres* in humans). This afferent class responds to non-noxious touch with a predilection for slow-moving, low-force, stroking stimuli such as gentle brushing [8–10]. It was shown that CLTMRs exhibit an inverted U-shaped (negative quadratic) response curve to single strokes of graded brushing velocities with peak discharge occurring at 1.0–10.0 cm/s. Conversely, the activity in myelinated afferents exhibited a linear relationship with stimulus velocity. Interestingly, the subjective ratings of perceived pleasantness also followed an inverted U-shaped pattern in relation to brushing velocity. Based on a correlation between neural discharge (impulse/s) and perception, it was concluded that CLTMRs mediate positive affective or pleasant touch [11]. However, it is also noteworthy that the use of a scale with the endpoints ‘unpleasant’ (-10) and ‘pleasant’ (+10) meant that the subjects reported low-velocity brushing (0.1 cm/s) in the negative/unpleasant range [11]. Whether this effect could be attributed to large- or small-fibre function, or the need for temporal summation, is a matter for conjecture. However, such bimodal association has been reinforced in recent work demonstrating that variation in the temperature of skin-stroking outside the thermal neutral zone can decrease the positive affect and enhance the negative affect [11,12]. In our previous psychophysical studies, we tested the effects of gentle brushing at CLTMR-optimal speeds of 1.0 and 3.0 cm/s–using the same robotic device for brushing as used in the aforementioned pleasant-touch work (and in current study)–on a clearly perceptual, stable level of ongoing muscle pain. We found that the otherwise non-painful brushing stimuli–applied for 30 s–generated a stimulus-locked exacerbation of the overall pain intensity, i.e. allodynia. This effect was elicited whether the myelinated fibres were conducting or not, thereby suggesting a role of low-threshold C fibres in allodynia [13,14]. However, other studies have hypothesised a rather indirect role of CLTMRs in pain processing by way of the malfunction of the pleasant-touch system [15].

In the current study we explored the following questions: What types of peripheral afferent fibres mediate pleasant and unpleasant tactile sensations? What are the effects of normally pleasant and unpleasant stimuli on tonic muscle pain? We opted for two types of test stimuli with different spatio-temporal properties: stroking stimulus using velvet or sandpaper at slow to moderate speeds; focal vibrotactile stimulus at low or high frequency. The interplay between affective processing and pain modulation was tested by applying both positive and negative affective stimuli during ongoing muscle pain, which was induced, and maintained, by a continuous infusion of hypertonic saline. All interactions were retested following a conduction blockade of myelinated fibres by compression. It was hypothesised that, following the induction of muscle pain, the *unpleasant* stimuli would manifest as *allodynia* while the *pleasant* stimuli would produce *analgesia*. Furthermore, it was hypothesised that the affective dichotomy and its subsequent modulatory effects on pain would remain preserved following the blockade of myelinated fibres.

## Methods

Healthy human subjects (*n* = 10; 3 females and 7 males) with no known musculoskeletal disorders or neuropathies participated in this study. This study was approved by the Human Research Ethics Committee (approval number: H9190) of the University of Western Sydney and complied with the principles of the revised Declaration of Helsinki. Informed written consent was obtained from each subject before commencing the experiment. All subjects were naïve to the aims and objectives of the study. In all experiments, subjects sat comfortably on a chair with both legs supported and extended for easier access to the tibialis anterior (TA) muscle. The muscle was palpated during active inversion of the foot and dorsiflexion of the ankle joint for identification of its anatomical boundaries.

In order to determine the contribution of different fibre classes to the affective (pleasant-neutral-unpleasant) components of tactile perception (***Experiment I***) and subsequently to pain modulation (***Experiment 2***), two types of stimuli–***stroking stimuli*** of varying textures (velvet and sandpaper) and focal ***sinusoidal*** vibration (low- and high-frequency)–were applied in both experiments while all nerve fibres were intact and following the blockade (***Compression***) of myelinated afferents. As with our earlier work [13], a two-compartment model was adopted: pain was induced in the TA muscle, and tactile/affective stimuli were applied to the overlying skin. This was aimed at avoiding any ambiguity as to whether the change in pain perception during concurrent innocuous stimulation reflected an altered responsiveness of cutaneous nociceptors or an altered integration of inputs at the central level, i.e. peripheral or central sensitisation.

Stroking stimuli were applied using a robotic device known as Rotary Tactile Stimulator (RTS: Dancer Design, UK). This device has been used extensively in CLTMR research to study their responsiveness to graded brushing velocities. For details, see [11]. In the current study, sandpaper (300 Grit) and velvet fabric–length 4 cm and width 3 cm–were attached to the manipulandum that swept across the skin surface (overlying TA) along a proximo-distal axis. These stimuli were applied at stroking velocities of 0.1, 1.0 and 10.0 cm/s, and a calibrated normal force of 0.4 N. Stroking velocities of 1.0 and 10.0 cm/s were chosen because they have been shown to produce a pronounced discharge in the CLTMRs; an effect that correlated with touch pleasantness ratings. Conversely, 0.1 cm/s was chosen because of its apparent capacity to elicit an unpleasant sensation [11]. Given the constraints of time following the induction of muscle pain, it was not feasible to test more than three stroking velocities. For the same reason, only a single stroke was applied per stimulus. Each stimulus combination was tested in triplicates, and applied in a random order to the same region of skin.

Sinusoidal vibration was applied to the skin overlying the TA using a circular Perspex (Plexiglas) probe with a 4-mm diameter tip. The probe was positioned perpendicular to the skin surface ∼15 cm distal to the tibial tuberosity and ∼1.5 cm lateral to the anterior border of tibia [13]. The probe was attached to a feedback-controlled mechanical stimulator. The frequency (20 and 200 Hz) and amplitude (200 μm) parameters of the stimuli have been previously used to study the discriminative aspects (localisation, intensity and frequency) of the classical, large-fibre-mediated tactile sensations but have not been systematically used to quantify affective responses [16–18]. However, based on our previous work in the cat, it is deducible that a stimulus of this sort can also activate low-threshold small-diameter fibres, including C fibres [19]. Likewise, the stimulus duration (30 s) was selected on the basis of previous observations where the onset of allodynic responses to C-fibre activation was delayed by ~10–15 s [13,14]. Low (20 Hz) and high (200 Hz) frequency stimuli were presented three times in a randomised order.

### Experiment 1: Affective responses

In *Experiment 1*, the affective qualities of stroking and vibrotactile stimuli were tested in 10 subjects using a Positive Affect and Negative Affect Scale **(*PANAS*)**. The magnitude of the affect was measured on a visual analogue scale ranging from 0 to 10. The scale was anchored by the following descriptors: Most Unpleasant (0); Neutral (5); Most Pleasant (10). All data were plotted as the percentage change from the unstimulated resting or neutral state (PANAS = 5).

In addition to PANAS, subjects were also provided a Visual Analogue Scale for Pain (***VAS***), which had a range from 0 (no pain) to 10 (worst pain). While the VAS was included for the pain-modulation observations (*Experiment 2*, see following text), it was nonetheless administered in *Experiment 1* with the aim of confirming that our affective stimuli themselves were perceived as non-painful by all participants (VAS = 0).

Based on prior experiments [13,14], an inter-stimulus interval of 45 s was used for both stimulus types (vibrotactile and stroking) in order to allow recovery of the skin and avoid adaptation of the neural system. This is consistent with the proposed recovery time for CLTMR function in animals (~30 s), and conforms to the stimulation interval followed in human psychophysical and microneurography studies [11,13,20,21]. To eliminate any effects of auditory and visual cues on subject responses, white noise was delivered through headphones and their vision was obscured.

### Experiment 2: Pain modulation

In *Experiment 2*, the effects of stroking and vibration on muscle pain were explored by infusing 5% hypertonic saline (HS; AstraZeneca Pty Ltd, North Ryde, NSW, Australia) into the TA. The HS was administered by inserting a 25 G butterfly cannula through the skin, ~6 cm distal to the tibial tuberosity, which was connected to an infusion pump (model 55–2226, Harvard Apparatus, Holliston, MA, USA). The hypertonic saline was infused (150–200 μl/min) into the TA in order to maintain a clearly perceptible, stable baseline pain for the duration of the infusion (∼15–20 min). All subjects were asked to report on the VAS whether stroking (velvet and sandpaper) or vibration (low and high frequency) increased, decreased or had no effect on the overall pain intensity.

### Nerve conduction blocks

In compression block experiments, a metal bar was placed distal to the ischial tuberosity in order to apply compression to the sciatic nerve. Somatosensory sensibility was tested within and beyond the innervation territory of sciatic nerve in order to compare and confirm the progression of the block. Myelinated blockade was confirmed by the loss of detection of vibration and cold within the affected region. Vibration sense was tested using the parameters of our test stimuli (20 and 200 Hz, 200 μm). Cold sense was tested by applying a ~15°C brass rod to the skin for 5 s. The preservation of warm sensibility (detection of a ∼40°C brass rod) was taken as confirmation for the intactness of C fibres [22–25]. Additionally, these stimuli were applied to the skin overlying the medial aspect of leg (innerved by femoral nerve), and the contralateral leg, in order to compare sensibilities across the affected and intact areas. Once the block had taken effect, the affective (no experimental pain, *Experiment 1*) and pain-modulatory (during HS, *Experiment 2*) effects of stroking and vibration were retested in separate experimental sittings.

### Statistical analysis

Data are presented as mean ± standard error of the mean (± SEM). In each individual, the responses to stroking and vibration were expressed as an absolute percentage change in affective rating (PANAS, *Experiment 1*) or pain rating (VAS, *Experiment 2*) immediately preceding tactile stimulation. Triplicate trials were treated as independent, sequential events: as triplicate trials of each stimulus combination were statistically indistinguishable, attesting to the reproducibility of the evoked effects, triplicate responses were pooled (within subjects) and averaged across all subjects. Significant changes were detected using 2-way repeated measures analysis of variance (RM-ANOVA) [26]. Where significant differences were indicated (*P* < 0.05), individual groups (control vs. compression) were compared using a Bonferroni correction multiple comparison test. All statistical comparisons were made using Prism 6 (GraphPad Software Inc., La Jolla, CA, USA).

## Results

### Stroking stimuli

When all fibres were intact, subjects readily distinguished between velvet and sandpaper based on their distinct sensory qualities and never reported them as painful (VAS = 0). However, following compression block, while the subjects could no longer perceive the sensory-discriminative aspects of the tactile stimuli such as onset, motion and pressure–consistent with the blockade of myelinated fibres–they were able to ascribe an affective rating to them. Akin to the intact condition, neither of the stimuli was perceived as painful (VAS = 0) following compression nor were there any visible signs of skin abrasion (e.g. redness) at the stimulation site. In all trials, the affective rating returned to the neutral level (PANAS = 5) before the next trial.

### Velvet

**Fig 1:**
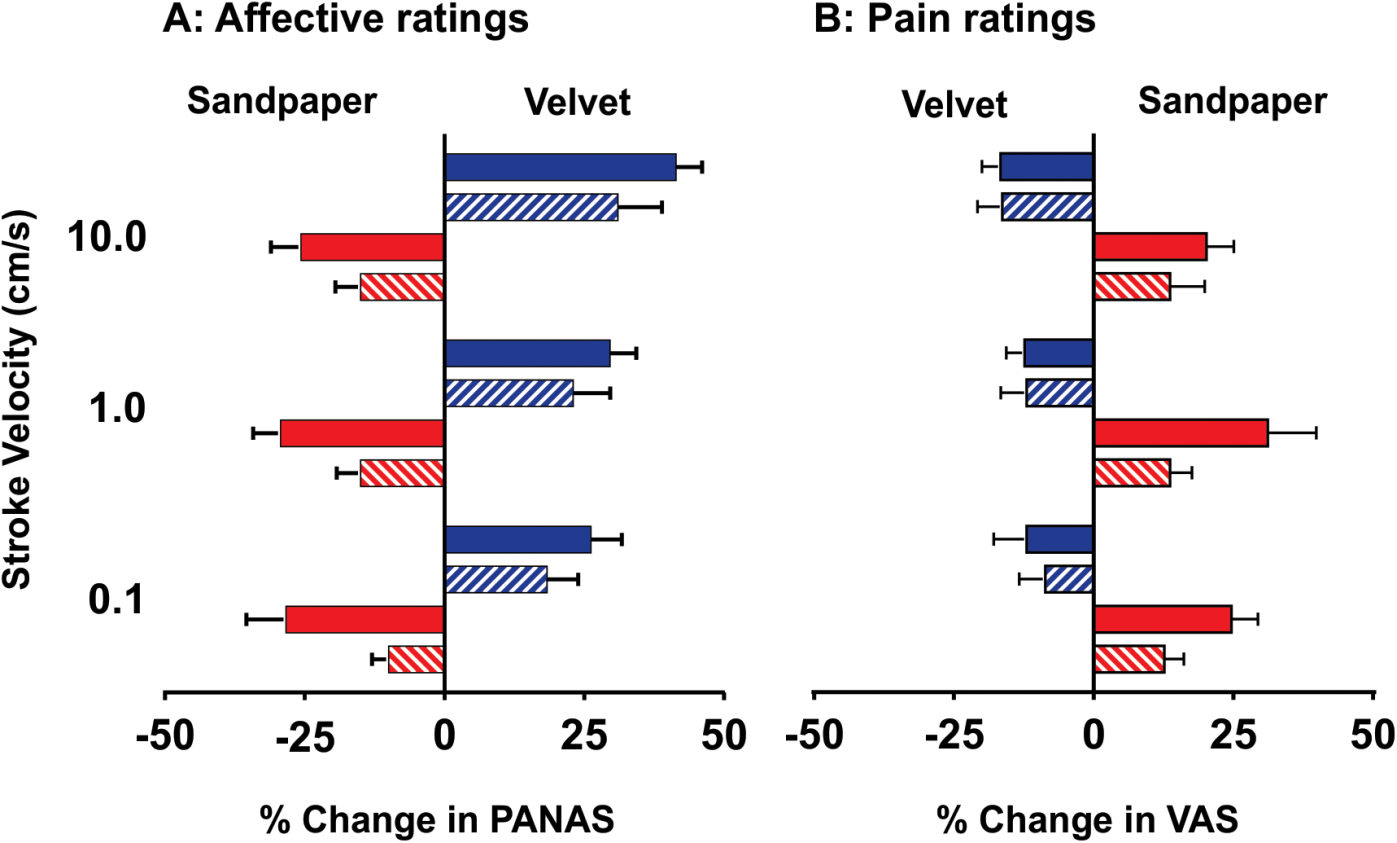
Affective responses (A) to stroking stimuli and their modulatory effects on muscle pain (B) prior to and following compression (mean ± SEM, *n* = 10). **A.** In response to stroking with velvet (blue bars), subjects reliably reported a pleasant (positive affective) quality, whereas an unpleasant (negative affective) quality was reported in response to stroking with sandpaper (red bars). These opposing effects were observed while all fibres were intact (solid bars) and following compression block of myelinated fibres (hatched bars). **B.** Following the induction of muscle pain, velvet-stroking reduced the overall pain intensity (analgesia), whereas sandpaper-stroking increased the overall pain intensity (allodynia). The analgesic and allodynic effects of velvet- and sandpaper-stroking persisted following the compression block of myelinated fibres (hatched bars).

In *Experiment 1*, all subjects reported stroking with velvet fabric as being pleasant (Fig 1A) over the range of test stimuli (0.1, 1.0 and 10.0 cm/s). During stroking with each velocity a reproducible sense of pleasantness or positive affect (*0*.*1 cm/s*: 26.2 ± 5.5%; *1*.*0 cm/s*: 29.6 ± 4.7%; *10*.*0 cm/s*: 41.4 ± 4.7%) was reported relative to the absence or neutrality of affect (PANAS = 5 or 100%) whilst the stimulus was in contact with the skin but remained stationary. Following compression, although the subjects were unable to perceive the discriminative aspects of the moving stimulus (i.e. the onset, pressure or textural properties of velvet), they could ascribe pleasantness to velvet-stroking (*0*.*1 cm/s*: 18.3 ± 5.6%; *1*.*0 cm/s*: 23.0 ± 6.6%; *10*.*0 cm/s*: 31.0 ± 7.9%). Importantly, pleasantness ratings were not significantly different between the intact and compression conditions (RM-ANOVA: F = 1.09; *P* = 0.32). While finely graded velocity differences were not observed between the three velocities tested, a significant difference was observed in the pleasantness ratings when comparing the extreme velocities (0.1 cm/s vs. 10.0 cm/s; *P* = 0.02) in the intact condition, but this was not evident following compression (*P* > 0.05).

Following the induction of muscle pain (*Experiment 2*, Fig 1B), stroking with velvet evoked suppression (analgesia) of the overall pain. This effect was stimulus-locked and short-lasting, as the pain intensity returned to levels immediately preceding stroking during the inter-stimulus interval. The *analgesic* effects of velvet-stroking were observed both prior to (*0*.*1cm/s*: -12.0 ± 5.9%; *1*.*0cm/s*: -12.3 ± 3.3%; *10*.*0cm/s*: -16.7 ± 3.3%) and following compression blockade (*0*.*1cm/s*: -9.7 ± 4.6%; *1*.*0cm/s*: -12.0 ± 4.6%; *10*.*0cm/s*: -16.3 ± 4.4%). Importantly, the stroking-evoked changes were not significantly different between the intact and compression conditions (RM-ANOVA: F = 0.04; *P* = 0.84). Furthermore, no significant differences emerged as a function of stroking velocity (RM-ANOVA: F = 2.26; *P* = 0.13).

### Sandpaper

In *Experiment 1*, all subjects reported stroking with sandpaper as evoking a negative affect (i.e. being unpleasant) but non-painful (Fig 1A). Although stroking with sandpaper was reported as unpleasant over the range of test stimuli, no significant differences were observed as a function of velocity (all fibres intact; *0*.*1 cm/s*: -28.3 ± 7.1%; *1*.*0 cm/s*: -29.3 ± 4.9%; *10*.*0 cm/s*: -25.7 ± 5.4%; RM-ANOVA: F = 0.53; *P* = 0.60). Following compression, although the effect sizes were significantly reduced relative to the intact condition, the subjects retained the capacity to attribute an unpleasant quality to the stimulus (*0*.*1 cm/s*: -10.0 ± 3.0%; *1*.*0 cm/s*: -15.0 ± 4.3%; *10*.*0 cm/s*: -15.0 ± 4.5%; RM-ANOVA: F = 9.69; *P* < 0.02).

Following the induction of muscle pain (*Experiment 2*, Fig 1B), stroking with sandpaper evoked a reproducible increase in the overall pain intensity from a steady pain rating observed prior to stroking across all three stimulation velocities (all fibres intact; *0*.*1 cm/s*: 24.7 ± 4.8%; *1*.*0 cm/s*: 31.2 ± 8.7%; *10*.*0 cm/s*: 20.2 ± 4.9%). No systematic differences were observed as a function of stimulus velocity (RM-ANOVA: F = 0.70; *P* = 0.51). Following compression, all subjects reliably reported increases in the overall pain during stroking with sandpaper (*0*.*1 cm/s*: 12.7 ± 3.5%; *1*.*0 cm/s*: 13.7 ± 3.9%; *10*.*0 cm/s*: 13.7 ± 6.2%; RM-ANOVA: F = 19.24; *P* = 0.002). The only significant reduction in effect size (44%) between the intact and compression conditions was observed at 1.0 cm/s (*P* = 0.0417).

### Focal vibration

**Fig 2:**
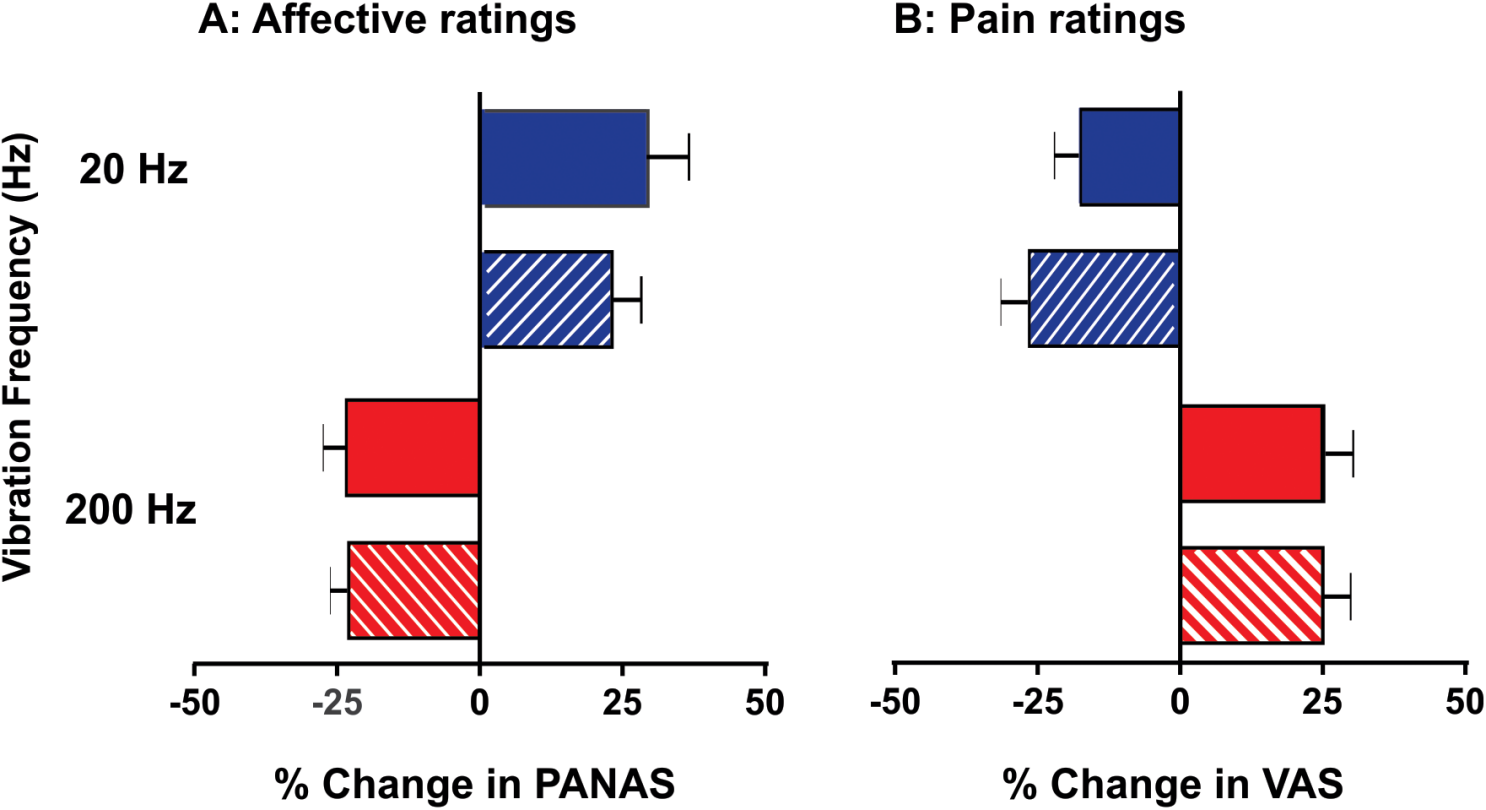
Affective responses (A) to vibrotactile stimuli and their modulatory effects on muscle pain (B) prior to and following compression (mean ± SEM, *n* = 10). **A.** In response to low-frequency vibration (solid blue bars) subjects reliably attributed a pleasant quality, whereas an unpleasant quality was reported in response to high-frequency vibration (solid red bars). **B.** Following the induction of muscle pain, low-frequency vibration reduced the overall perception of pain (analgesia), whereas high-frequency vibration increased the overall pain (allodynia). The relationship between stimulation frequency, affective regard and pain modulation was preserved following the compression block of myelinated fibres (hatched bars).

When all fibres were intact, all subjects readily distinguished between the two vibrotactile stimuli in the low (20 Hz) and high (200 Hz) frequency range. Subjects were instructed to disregard the vibratory or discriminatory (frequency, intensity and location) aspect of the stimulus and focus on the affective attributes, that is, whether the stimulus evoked a pleasant or unpleasant sensation, or whether it was devoid of any affective quality. All subjects reported low-frequency vibration as pleasant and high-frequency vibration as unpleasant (as rated on PANAS; Fig 2A). Neither of the stimuli was reported as painful (VAS = 0).

A two-way RM-ANOVA revealed that although the magnitude of the effects observed at 20 and 200 Hz was comparable, the sign or direction of the effects was opposing. Furthermore, the amplitude of the effects did not differ between the intact and compression conditions (RM-ANOVA: F = 0.40; *P* = 0.54). Likewise, the opposing effects on muscle pain were statistically indistinguishable between the two conditions (RM-ANOVA: F = 0.0003; *P* = 0.99).

### 20-Hz vibration

In *Experiment 1*, all subjects perceived the 20-Hz vibration as being pleasant, which was reported as a positive change in the PANAS rating. Fig 2A shows the pooled mean data of triplicate responses for the intact condition (28.6 ± 7.3%) and following compression (22.4 ± 5.1%). In each trial, the affective ranking returned to the neutral level before the next trial.

Following the induction of muscle pain (*Experiment 2*, Fig 2B), all individual responses and the pooled mean data showed that the 20-Hz vibration evoked a reproducible reduction in the overall pain both prior to (-17.7 ± 4.2%) and following compression (-26.1 ± 4.7%). In the absence of any superimposed vibration, the HS-induced muscle pain did not vary significantly (*P* > 0.05) throughout the experiment or between the intact and compression conditions. These observations demonstrate that low-threshold C-fibre inputs can elicit analgesia during ongoing muscle pain.

### 200-Hz vibration

In *Experiment 1*, all subjects reported the 200-Hz vibration as being unpleasant but not painful (Fig 2A). In each case, the overall affective rating was reproduced on at least three occasions both prior to (-22.8 ± 4.0%) and following (-22.4 ± 3.0%) compression block.

Following the induction of muscle pain (*Experiment 2*, Fig 2B), the pooled mean data show that 200-Hz vibration evoked an increase in the overall pain intensity both prior to (24.4 ± 4.9%) and following (24.4 ± 4.8%) compression block. Hence, a stimulus perceived as unpleasant (not painful) manifested as allodynia during ongoing muscle pain. Both negative affect and pain exacerbation persisted while the myelinated fibres were conducting or not, thereby suggesting a role of low-threshold C fibres in mediating these responses.

## Discussion

In this study we provide psychophysical evidence that both positive (pleasant) and negative (unpleasant) affective tactile attributes are reliably ascribed to ‘everyday’ textural surfaces as well as to less familiar stimuli such as sinusoidal vibration. Furthermore, these affective judgments were preserved following the conduction block of myelinated fibres, indicating that affective touch sensations can be sustained by C-fibre inputs alone. Indeed, this was most clearly demonstrated with vibrotactile stimuli where the subjects were unable to detect the discriminative (onset, intensity and frequency) aspects of vibration following the compression block, yet they could reliably ascribe positive and negative affections to 20-Hz and 200-Hz vibration even though the order of stimuli was randomised. Such stimulus fidelity was also observed following the induction of muscle pain: those stimuli perceived as pleasant (velvet and 20-Hz vibration) reduced the overall perception of pain, whereas those perceived as unpleasant (sandpaper and 200-Hz vibration), but not painful, increased the overall pain. The modulatory effects on pain (*i*.*e*. allodynia and analgesia) persisted when the myelinated fibres were blocked, thereby suggesting that these effects can be mediated by C fibres alone. These results not only confirm our earlier findings that CLTMRs can mediate the allodynic effect of 200-Hz vibration during ongoing muscle pain [13,27], but also provide new evidence that both allodynia and analgesia can be subserved by cutaneous afferents within the C-fibre range. Consistent with our earlier work, we elected to use a two-compartment model, in which pain was induced within the muscle and cutaneous responses tested in the overlying skin, in order to ensure that the observed changes in affective and nociceptive processing were the result of central interaction rather than a change in primary afferent responsiveness within the skin.

In an attempt to disentangle the contribution of different afferent fibre classes for velvet and sandpaper experiments, we selected a range of stimulus velocities including those that have been shown to be optimal for CLTMR activation [11]. As noted in the Introduction, the presence of comparable inverted U-shaped tuning curves for the afferent discharge and the corresponding psychophysical judgments have been previously reported in support of the role of CLTMRs in affective judgments. In our experiments, the use of two textures (sandpaper and velvet), although failing to reveal any finely graded velocity-dependent effects, did reveal opposing texture-based effects that were reproducible in both intact and compression conditions. How the complex sensory or affective attributes are encoded in the response of an individual or population of C fibres remains unclear. However, the present data demonstrates that both positive and negative attributes can be reliably detected in the presence of unmyelinated fibres with the myelinated fibres blocked.

Even though CLTMRs respond to static touch [9,28], static contact of velvet or sandpaper with the skin was not sufficient to evoke a distinct affect, therefore it appears that the progressive recruitment of multiple units across the skin surface (as with a stroking stimulus) is required in order to generate an affective qualia. In the current study, the use of a controlled mechanical device (RTS) to deliver high-precision (in terms of force, velocity and direction) stimuli to the skin, namely the soft pile of velvet fabric versus the hard grains of sandpaper, resulted in opposing affective qualities. Such considerations are evident in the earlier work where delivering controlled stimuli of varying textures resulted in modulation of the affective rating [29]. One way to explain the opposing affects evoked by moving stimuli is to postulate that each stimulus sets up a differential pattern of afferent discharge based on the spatial distribution of textural elements. In addition, the effects of varying surface textures may well be influenced by skin compliance and frictional forces at the skin-stimulator interface, thus explaining, in part, the lack of a distinct affective quale with static mechanical contact. Studies on compliance encoding have argued for a critical role of large-diameter fibres such as slowly adapting afferents. However, the role of affective coding in this context remains largely unexplored [30,31]. Previous studies [29,32] have conjectured about the involvement of low-threshold C mechanoafferents in affective judgments, but they did not test the contribution of C fibres by using a conduction blockade of myelinated fibres. Our experiments confirmed that subjects could not only detect the affective attributes of touch following the blockade of myelinated fibres, but also reliably discriminate between the opposing affective stimuli.

The demonstration of opposing frequency-dependent affective responses when vibratory stimuli were applied to a fixed point on the skin highlights the complexity of the afferent coding of affect and introduces the possibility of coding strategies based on differential afferent class contributions and/or the pattern of impulse activity initiated at the fixed site of stimulation. In contrast, in the velvet/sandpaper task, judgments appeared to be based on complex spatio-temporal recruitment of afferent activity as the texture was moved across the skin surface. Furthermore, while both tasks (texture and vibration) can generate positive and negative affect, it remains unclear whether the presence of comparable affective responses arises due to complex patterns of spatial-temporal convergence at a spinal or cortical level. However, our observations that the perceived quality of the affect (positive or negative) attributed to a stimulus can reliably predict the modulatory effects on muscle pain (increase or decrease) suggests that both affect and pain may well be processed within closely linked circuits in the central nervous system.

Studies examining the affective processing in a large-fibre deafferented patient revealed a pattern of activation in the insular cortex, and deactivation in the somatosensory, motor, anterior cingulate, parietal association and prefrontal cortices as well as thalamus [10,33]. The deactivation of areas implicated in pain processing has been used to argue for a role of CLTMRs in the suppression of pain. Intriguingly, remarkably similar areas of activation were observed when brushing was delivered to one’s own skin as well as others’ skin surface, suggesting that ‘empathetic touch’ or the associated affect can generate comparable patterns of cortical activation [34]. Furthermore, the coupling of multimodal stimuli has shown that high-saliency affective stimuli such as disgusting odours can decrease touch pleasantness [35].

The proposition that CLTMRs normally supress ‘pain’ inputs needs to be broadened in light of our previous observations where the expression of allodynia–evoked by 200-Hz vibration and gentle brushing at CLTMR-optimal speeds–in rapid-onset, delayed-onset and chronic pain conditions remained preserved following the preferential blockade of myelinated fibres but was abolished following the preferential blockade of C fibres in the skin [13,14,25,27]. Furthermore, in the current study, we have shown that, following the blockade of myelinated nerves, subjects could reliably detect affective stimuli, which in turn predicted their modulatory effects on muscle pain. Moreover, a unimodal role cannot completely explain the reported correlations between afferent recordings and psychophysical observations that included both positive and negative affective ratings [11]. To resolve this conundrum, further research is warranted into the coding mechanisms of low-threshold C fibres. Consistent with recent molecular/genetic studies where heterogeneity within the CLTMR population has been reported, further investigation into these functionally undefined subtypes is warranted [36,37].

Molecular studies have identified a host of target molecules that, in addition to serving as markers of different fibre classes, may play a critical role in defining the contribution of unmyelinated fibres to synaptic processing and pain modulation [36,38]. For example, CLTMRs project to the inner part of lamina II, a region implicated in the transition from acute to persistent pain and injury-induced mechanical allodynia [39,40]. In addition, CLTMRs co-express the pro-nociceptive glutamate and the analgesic TAFA4 protein [36,41]. The complexity of CLTMR contributions to synaptic processing is further highlighted by the expression of GINIP, a Gα-inhibitory interacting protein, which normally enhances the level of presynaptic inhibition in both TAFA4-expressing CLTMRs and Mas-related G-protein-coupled receptor D-positive (MrgprD+) neurons [38]. In both cases, the use of knock-out models has demonstrated that the loss of either molecule (GINIP/TAFA4) results in pronounced (and prolonged) mechanical hypersensitivity following injury. However, further work is required to determine how the balance between glutamate-mediated excitation, TAFA4 and GINIP-mediated processes can contribute to a shift between positive and negative affect, let alone the opposing modulatory effects observed during muscle pain in this study.

Recent animal work [37] showing that tactile (and cold) allodynia are dependent upon the expression of low-voltage T-type Cav3.2 channels in CLTMRs reinforces our hypothesis that this afferent class contributes to allodynia observed following acute muscle pain, delayed onset muscle soreness and in clinical subjects [13,25]. Beyond the demonstration that selective Cav3.2 knock-out in mice diminished tactile and cold allodynia following injury, the pharmacological blockade of Cav3.2 channels in wild type mice resulted in decreased responsiveness of CTLMRs (increased excitability threshold). Furthermore, in our recent work [42], we have demonstrated that the use of the same calcium channel antagonist abolished experimental cold allodynia in healthy human subjects–providing additional support for the role of CLTMRs in pain processing.

In conclusion, we have provided evidence that low-threshold cutaneous afferent fibres within the C-fibre range contribute to affective and pain processing. Affective tactile sensations, pleasantness and unpleasantness, were evoked using two types of test stimuli with different spatio-temporal properties. Importantly, we found that the affective attributes of tactile stimuli predicted their modulatory effects on pain. That is, the unpleasant stimuli evoked allodynia and the pleasant stimuli evoked analgesia. Following the blockade of myelinated fibres, although the sensory-discriminative aspects of touch were impaired, the capacity to perceive affective touch remained intact. Likewise, following the induction of muscle pain, the capacity of affective stimuli to evoke allodynia and analgesia was preserved regardless of whether the myelinated fibres were conducting or not. Further investigation is warranted into the characterisation of CLTMRs and the coding mechanisms underpinning their dichotomous role in affect-based modulation of pain.
